# Synaptic control of retinal ganglion cell survival and axon regeneration

**DOI:** 10.1186/s13024-026-00929-1

**Published:** 2026-01-28

**Authors:** Yuxuan Qiu, Qi Zhang, Jiahui Tang, Yunjie Cheng, Yuxin Wang, Zijie Wang, Xuehan Liu, Bing Zhang, Liyan Liu, Shilong Yu, Yangjiani Li, Zhe Liu, Fang Chai, Yehong Zhuo, Yiqing Li

**Affiliations:** 1 State Key Laboratory of Ophthalmology, Zhongshan Ophthalmic Center, Sun Yat-sen University, Guangdong Provincial Key Laboratory of Ophthalmology and Visual Science, Sun Yat-sen University, Guangzhou, 510060 China; 2 Zhongshan School of Medicine, Sun Yat-sen University, Guangzhou, 510080 China; 3 Department of Ophthalmology, Sun Yat-Sen Memorial Hospital, Sun Yat-Sen University, Guangzhou, 510120 China; 4 Shaanxi Eye Hospital, Xi’an People’s Hospital (Xi’an Fourth Hospital), Affiliated People’s Hospital of Northwest University, Xi’an, 710004 China

**Keywords:** Synapse, Retinal ganglion cells, Survival, Axon regeneration, Central nervous system

## Abstract

**Background:**

Injury to retinal ganglion cell (RGC) axons in neurodegenerative conditions like glaucoma leads to irreversible vision loss. A major therapeutic challenge is promoting RGC survival and axon regeneration. Canonical research focused on intrinsic neuronal growth capacity and the inhibitory central nervous system (CNS) environment, but overlooking the role of retinal synaptic communication.

**Main body:**

This review summarizes emerging evidence that retinal interneuron-to-RGC synaptic connections are both structurally and molecularly dysregulated following RGC axon injury. Such synaptic plasticity critically regulates RGC survival and regenerative capacity, at least partly by orchestrating intrinsic repair programs. We then address two central unresolved questions: first, what are the specific molecular pathways that alter this interneuron-to-RGC signaling after injury, and second, how do glial cells participate in this transsynaptic dysregulation. Finally, we evaluate the translational potential of these findings, including the identification of biomarkers and the development of novel neuroprotective strategies that target synaptic connections.

**Conclusion:**

Synaptic communication is a fundamental regulator of RGC fate after injury. Understanding synaptic dysregulation and the mechanisms involved is essential for developing new synapse-targeted strategies to monitor progression of neurodegenerative diseases and promote neural repair.

## Background

Vision is essential to human perception. Retinal ganglion cells (RGCs) are the principal output neurons of the retina, and their axons form the optic nerve, the sole pathway transmitting visual information from the eye to the brain. Notably, the axons of these central nervous system (CNS) neurons are particularly susceptible to injury [1]. In adult mammals, progressive RGC loss and axon degeneration are irreversible and commonly occur after RGC axon damage across myriad neurodegenerative conditions, including glaucoma and other optic neuropathies [2–4].

To elucidate the molecular pathobiology of neurodegeneration following RGC axon injury, researchers have widely utilized animal models such as optic nerve injury (ONI; including optic nerve crush and transection) and ocular hypertension (OHT) [5]. Studies using these models have established that enhancing intrinsic neuronal growth programs while counteracting inhibitory cues in the CNS microenvironment can drive robust neuronal preservation and axonal regrowth (reviewed in [1–4, 6]). Comparatively, the role of synaptic signaling from retinal interneurons to RGCs remains largely uncharacterized.

**Fig. 1:**
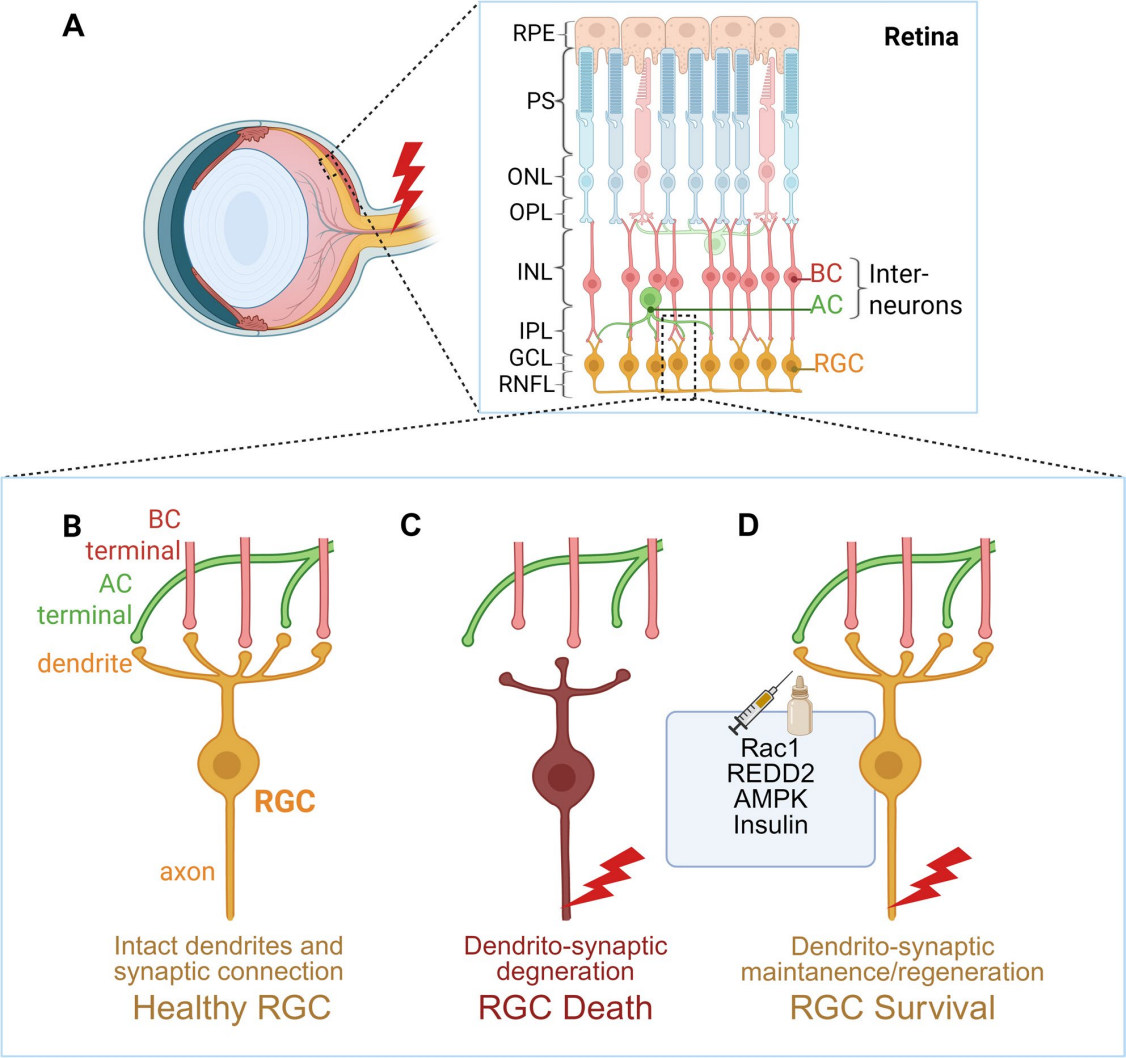
Anatomy of interneuron-to-RGC synapses and dendrito-synaptic plasticity after RGC axon injury. RGC axons are damaged by diverse optic nerve insults (**A**, red lightening symbol). BCs and ACs form synapses onto RGCs in the IPL (**A**). Under homeostatic conditions, RGCs extend fully arborized dendrites into the IPL, receiving synaptic signals from BC and AC terminals (**B**). Following RGC axon injury, these connections deteriorate, with rapid dendritic retraction that precedes RGC death (**C**). Therapeutic interventions that preserve dendritic structure, such as Rac1 activation or REDD2 knockdown, or that promote dendritic regrowth, such as AMPK knockdown or insulin eye drops, increase RGCs resilience to axon injury (**D**). Abbreviations: AC, amacrine cell, shown in green; AMPK, adenosine monophosphate-activated protein kinase; BC, bipolar cell, in red; GCL, ganglion cell layer; INL, inner nuclear layer; IPL, inner plexiform layer; ONL, outer nuclear layer; OPL, outer plexiform layer; PS, photoreceptor segment; REDD2, regulated in development and DNA damage response 2; RGC, retinal ganglion cell, in yellow; RNFL, retinal nerve fiber layer; RPE, retinal pigment epithelium. Created in https://BioRender.com

RGCs are embedded within retinal circuits, extending dendrite arbors into the inner plexiform layer (IPL), where they receive synaptic inputs from bipolar cells (BCs) and amacrine cells (ACs) [7] (Fig. 1A). Seminal works demonstrated that optic nerve damage elicits early dendritic degeneration in RGCs accompanied by retinal synaptic dysfunction [8, 9]. Notably, these structural and functional alterations can precede overt RGC death and are associated with substantial impairment of visual function [8, 9]. Furthermore, accumulating evidence underscores the importance of retinal synaptic communication as a crucial regulator of RGC survival and regenerative competence after axon injury. Specifically, interventions that preserve or restore dendritic architecture and reestablish synaptic connectivity enhance survival of axotomized RGCs [10–14], and synaptic signals from BCs and ACs impact RGC fate under the cellular stress imposed by optic nerve damage [15–18]. Collectively, these findings highlight the previously underappreciated transsynaptic mechanisms as critical determinants of neuroprotection and axon regeneration.

Significant gaps persist in our understanding of transsynaptic communication in the context of RGC axon injury. First, the interneurons providing synaptic signals to RGCs are anatomically distant from the primary lesion site, raising questions about how they detect and respond to remote injury. Axon-to-soma injury signaling [19] and retrograde transsynaptic communication [20] are potential mechanisms, but their specific roles in the pathophysiology of RGC retinal synapses remain largely unexplored. Second, glial cells, which are in proximity to and actively modulate synapses [21], may play a critical role in post-lesion transsynaptic signaling. However, their precise involvement in this process remains elusive. Addressing these knowledge gaps is essential to advancing our understanding of neuronal responses to injury and to guiding the development of novel therapeutic strategies.

In this review, we summarize recent advances in understanding how synaptic drive from retinal interneurons influence RGC survival and axon regeneration. We then critically evaluate unresolved questions in post-injury transsynaptic signaling, with an emphasis on how injury signals are transmitted from RGC axons to interneurons. In addition, we discuss glial modulation of transsynaptic communication, particularly at synapses between interneurons and RGCs. Finally, we summarized advances in translational studies that are turning transsynaptic injury mechanisms into clinically viable biomarkers and therapeutics. Our goal is to provide an integrated overview of synaptic remodeling following RGC axon injury and to identify key knowledge gaps that should guide future research into synapse-driven neural repair.

## Dysregulated interneuron-to-RGC synaptic signals after RGC axon injury

In the mammalian retina, BCs and ACs constitute the principal synaptic inputs to RGCs [7] (Fig. 1A). The cell bodies of these interneurons are predominantly located in the inner nuclear layer, although displaced ACs are also present within the ganglion cell layer [22]. BCs relay visual signals from photoreceptors in the outer retina to RGCs in the inner retina, while ACs engage in complex synaptic interactions with both BCs and RGCs. BCs and ACs extend neurites into the IPL, where they synapse onto RGC dendrites [23]. Collectively, signals from BCs and ACs to RGCs lay the foundation of precise visual encoding in the retina.

Following axon injury to RGCs, their retinal synaptic connections undergo rapid remodeling (Table 1). This synaptic plasticity significantly influences RGC survival and regenerative capacity. Therefore, targeting interneuron-to-RGC synapses represents a promising therapeutic avenue to promote neuroprotection and optic nerve regeneration.

### RGC dendrites

As specialized neuronal compartments that extend into the IPL, RGC dendrites serve as the principal sites for receiving synaptic signals from BCs and ACs (Fig. 1B). In multiple glaucoma models, RGC dendrites exhibit pronounced retraction before overt neuronal loss [24–29] (Fig. 1C), a phenomenon that parallels the synaptic pruning observed broadly across the nervous system (Box 1).

Preservation or restoration of RGC dendritic architecture is associated with enhanced neuronal survival (Fig. 1D). Manipulation of Rho-family GTPase Rac1 [10] or knockdown of regulated in development and DNA damage response 2 (REDD2) [11] maintains dendritic structure and improve RGC survival after ONI. In OHT models, activation of adenosine monophosphate-activated protein kinase (AMPK) contributes to dendrite retraction by suppressing mTOR signaling, whereas reducing AMPK activity limits dendrite pathology and attenuates RGC loss [13]. Notably, administration of insulin eye drops induces robust RGC dendrite regeneration, even after manifest dendritic retraction following ONI- or OHT-induced axon injury [12, 14]. The regenerated dendrites restore BC-to-RGC excitatory synapses and protect against degeneration through insulin receptor signaling in RGCs [12, 14]. Also of interest, despite marked dendritic retraction, RGC excitability shows a transient increase at approximately two weeks after OHT induction [28]. This effect is not attributable to with a-amino-3-hydroxy-5-methyl-4-isoxazolepropionic acid receptor (AMPAR)-mediated glutamatergic signaling, but instead depends on voltage-gated activity [28], underscoring that RGC responses to altered synaptic input are regulated by both structural remodeling and intrinsic excitability.

These observations suggest that axon injury-induced dendritic remodeling may be a key determinant of RGC fate. However, many interventions that preserve or restore dendritic morphology are also broadly neurotrophic, which complicates the causal inference between synaptic connectivity and neuroprotection. Therefore, it remains unclear whether dendrite-preserving or dendrite-regenerating treatments enhance specific forms of neurotransmission, and how such changes influence RGC survival. The relationship between dendritic dynamics and axon regeneration likewise requires more detailed investigation. Notably, studies in zebrafish indicate an antagonistic interplay between axon and dendrite repair, with tight temporal constraints on the restoration of each compartment [30], as further discussed in prior reviews [31, 32].

**Box 1. Post-axotomy synaptic elimination in the nervous system**

Synaptic elimination following aervous system (PNS). In a seminal study, Blinzinger and Kreutzberg observed that within four days of facial nerve transection, microglial processes closely attached to motoneuron perikarya and dendrites, facilitating the removal of synaptic terminals from neuronal postsynaptic membranes [xotomy was first characterized in the peripheral n^33^]. This process, known as synaptic pruning, plays a critical role in remodeling of neuronal connections after injury [34], resembling developmental synapse refinement [35, 36]. Importantly, synapse elimination is selective. After axon injury, glutamatergic presynaptic boutons are preferentially removed over glycine- and GABA-containing ones [37]. This targeted stripping of excitatory inputs is thought to mitigate excitotoxicity, thereby promoting neuronal survival and axon regeneration [34, 38, 39]. These findings underscore the critical role of synaptic connections in neuron viability and regeneration in the PNS.

In the CNS, synapse elimination is also a common response to axon injury. Following spinal cord injury (SCI), both complexity and density of basal dendrites, key sites for receiving synaptic inputs, are markedly reduced [40, 41]. Similarly, shortly after CNS axon injury in vitro and in vivo, lesioned neurons exhibit dendritic spine loss, and receive heightened presynaptic excitatory signals along with diminished inhibitory inputs [42]. By 48 hours post-injury, presynaptic neurons demonstrate a significant increase in synaptic vesicle release rate at terminals facing postsynaptic membranes of their axotomized partners [42]. Studies in human SCI patients have also reported post-lesional changes in cortical neurotransmission [43, 44]. Given the rapid reorganization of synaptic architecture and signals following SCI, it is critical to determine whether these alterations influence neuroprotection and regeneration, as observed in ONI (section “Dysregulated interneuron-to-RGC synaptic signals after RGC axon injury”). In lamprey, SCI induces a sharp upregulation of GABA receptor subunit, with endogenous GABA signaling promoting neuronal survival and boosting axon regeneration via GABAB receptor activation [45]. In addition to SCI, traumatic brain injury also leads to rapid alterations in synaptic proteins and synapse loss, potentially impacting secondary injury progression [46].

Elucidating the mechanisms by which synapses are eliminated after axotomy offers important insights into the pathophysiology of a broad range of peripheral and central nervous system disorders. Increasing evidence indicates that immune synaptic crosstalk is a central driver of this process. In the PNS, major histocompatibility complex class I (MHC-I) has been identified as a key regulator of synaptic pruning [47–51]. Loss of MHC-I function disrupts the selective elimination of excitatory inputs while impairing inhibitory input preservation, leading to reduced axon regeneration [49]. Similarly, genetic ablation of CD3ζ, an essential component of MHC-I, leads to abnormal RGC dendrite motility and altered synaptic transmission during retinal development [52]. However, it remains to be determined whether and how MHC-I is involved in dendritic degeneration after axon injury in mature RGCs. Beyond MHC-I, other canonical immune pathways, including the complement cascade, also participate in post-axotomy synaptic remodeling. For instance, activation of the complement cascade has been implicated in microglia-mediated synaptic elimination following axon injury in both PNS [53, 54] and CNS neurons (section “Microglial cells”). Altogether, synaptic elimination is a widespread pathophysiological consequence of axon injury in both PNS and CNS.

### BC-derived glutamate

In addition to the structural plasticity of retinal synapses described above, researchers also examined specific interneuron-to-RGC signaling following RGC axon injury. Glutamate excitotoxicity, driven by pathological glutamate accumulation, was among the earliest proposed mechanisms to explain post-injury RGC degeneration, yet its contribution to disease progression remains debated [55, 56]. Initial studies reported elevated glutamate levels in the vitreous of patients with glaucoma and monkeys with experimentally induced OHT [57]. Subsequently, multiple groups have described either increased [58–60] or unchanged [61–65] glutamate levels across axon injury and ischemic models, spanning species that include rodents, nonhuman primates and humans (Table 1). Notably, these measurements were obtained from adjacent anatomical compartments, rather than directly from the retina itself (Table 1). Because physical separation and barriers such as the inner limiting membrane lie between the putative excitotoxic sites at retinal synapses and the sampled fluids, it is uncertain whether changes detected in these samples accurately reflect post-injury glutamatergic events occurring near BC-to-RGC synapses [56].

**Fig. 2:**
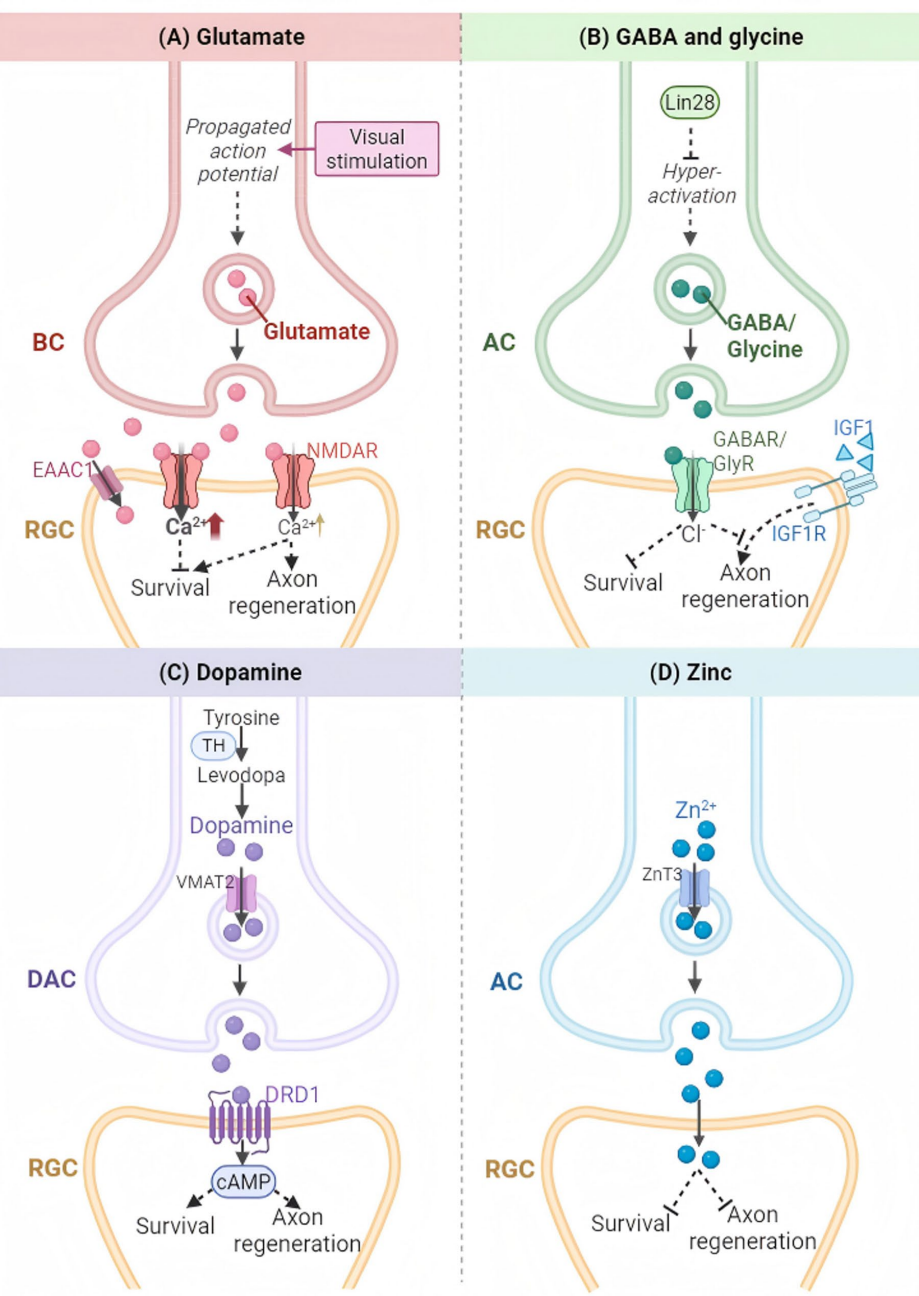
Synaptic signals regulate RGC survival and axon regeneration. Excessive glutamate contributes to RGC damage, while appropriate excitatory input is essential for RGC survival and axon regeneration (**A**). Inhibitory neurotransmitters, specifically GABA and glycine, released from ACs, bind to receptors on RGCs, inhibiting RGC survival and reducing responsiveness to IGF1-induced axon regeneration (**B**). Dopamine released from DACs specifically binds to DRD1 receptors on RGCs, promoting neuroprotection and enhancing axon regeneration (**C**). Elevated Zn^2+^ is transported into vesicle via ZnT3 in ACs and subsequently released to RGCs, where it impairs RGC survival and regenerative capacity (**D**). Abbreviations: AC, amacrine cell; Ca^2+^, calcium ion; DAC, dopaminergic amacrine cell; DRD1, dopamine receptor D1; EAAC1, excitatory amino acid carrier 1; GABA, γ-aminobutyric acid; IGF1, insulin-like growth factor 1; IGF1R, insulin-like growth factor 1 receptor; RGC: retinal ganglion cell; TH, tyrosine hydroxylase; VMAT2, vesicular monoamine transporter 2; Zn^2+^, zinc ion; ZnT3, zinc transporter 3. Created in https://BioRender.com

Despite ongoing debate regarding whether glutamate is pathologically elevated in vivo, its neurotoxic potential is well-documented. (Fig. 2A). In experimental settings, intraocular administration of glutamate or the glutamatergic receptor agonist N-methyl-D-aspartic acid (NMDA) is a well-established approach to elicit excitotoxic RGC degeneration [66–76]. Consistently, global deletion of glutamate transporters that remove extracellular glutamate, including glutamate/aspartate transporter (GLAST) and excitatory amino acid carrier 1 (EAAC1), produces glaucoma-like retinal degeneration [77], supporting a role for glutamatergic dysregulation in RGC pathology. Pharmacological blockade of NMDA receptors (NMDARs) has been reported to protect RGCs from several injury paradigms [73, 78–84]. However, the efficacy of NMDAR inhibition has not been uniform across studies. For instance, memantine, an uncompetitive NMDAR antagonist, provided limited structural and electrophysiological protection in nonhuman primate OHT models [85, 86], and oral memantine did not slow glaucoma progression in clinical trials (NCT00141882, NCT00168350) [87].

Importantly, glutamatergic signaling can exert divergent effects on RGCs in a context-dependent manner. While excessive glutamate is neurotoxic, appropriately regulated glutamatergic transmission is essential for normal synaptic function and may promote RGC survival and axon regrowth after injury. In line with this concept, axon regeneration can be boosted by visual stimulation [88, 89], which increases glutamate release from specific BC subtypes (Fig. 2A). Recently, Karen Chang and colleagues demonstrated in cultured RGCs that activity-induced neurite outgrowth is mediated by glutamatergic signaling [18]. Building on this, they developed a biocompatible material designed for sustained glutamate release, which promoted neuroprotection and axon regeneration in vivo following ONI [18].

Over the past decades, controversies regarding the pathophysiological contribution of glutamate have motivated a shift towards multicellular mechanisms that govern glutamate homeostasis, rather than focusing exclusively on RGC intrinsic responses [56]. Accordingly, most studies have emphasized how glial cells regulate and response to extracellular glutamate (reviewed in [90]). By contrast, BCs, the primary source of glutamatergic input to RGCs, remain comparatively understudied. A transsynaptic perspective is required to fully capture how glutamate signaling is regulated within injured visual circuits.

Consistent with this notion, BCs exhibit early changes after RGC axon injury. Specifically, early remodeling of BC presynaptic proteins and subtype-specific BC-RGC connectivity has been reported after transient OHT-induced RGC injury [91, 92]. In rod BCs (RBCs), protein kinase C α (PKCα) decreases before manifest RGC loss in NMDA-induced excitotoxicity, accompanied by reduced RBC activity [93]. The same study reported that ONI and acute OHT similarly reduced PKCα expression in RBC dendrites [93]. In addition, both ageing and acute OHT impair excitatory synaptic input from BCs to RGCs [29]. In chronic OHT, an increased in BC glutamatergic active zone has been inferred from elevated levels of the synaptic ribbon protein RIBEYE by approximately two weeks after OHT onset [28]. Meanwhile, glutamate transport via BC glutamate transporter 1 plays a key role in a multi-neuronal signaling pathway that facilitates RGC axon regeneration [94]. These findings collectively indicate that BC-to-RGC neurotransmission rapidly adapts to RGC axon damage and can modulate RGC responses across diverse injury contexts.

### AC-derived GABA and glycine

Inhibitory neurotransmitters from ACs play a critical role in regulating RGC survival and axon regeneration. Following ONI, ACs become hyperactivated within one day and maintain elevated activity for at least one week (Table 1). This hyperactivation leads to excessive release of inhibitory neurotransmitters, γ-aminobutyric acid (GABA) and glycine, which suppress RGC activity, exacerbate neuronal loss, and impair axon regeneration by reducing RGC responsiveness to insulin-like growth factor 1 (IGF1). Notably, AC-specific overexpression of Lin28 counteracts AC hyperactivation and alleviates transsynaptic inhibition of both RGC survival and IGF1 responsiveness [16]. Thus, targeting inhibitory signals may represent a promising strategy to promote neuroprotection and axon regeneration (Fig. 2B).

The impact of excitatory and inhibitory signals on RGC fate highlights the crucial role of neuronal activity. RGC activity appears to be closely linked to post-injury survival and the ability to regenerate [16–18, 28, 88, 89, 95–97]. This aligns with evidence that neuronal activity promotes neuron preservation and neurite outgrowth in other CNS neurons [98]. Therefore, restoring or enhancing neuronal activity can be a key strategy for unlocking the survival and regenerative potential of CNS neurons.

### AC-derived dopamine

Intriguingly, not all AC subpopulations are hyperactivated following RGC axotomy. A subset of wide-field ACs that release dopamine (DA), known as dopaminergic ACs (DACs) [99], exhibit decreased activity as early as one day after ONI, accompanied by diminished DA synthesis and release (Table 1). Restoring DA levels through intravitreal L-DOPA injection or direct DAC reactivation may represent a novel therapeutic strategy to promote neuroprotection and axon regrowth [17]. Moreover, RGC-specific overexpression of *Drd1*, a DA receptor enriched in resilient RGC subtypes, enhances regenerative effect of L-DOPA [17] (Fig. 2C). These findings underscore the multifaceted roles of ACs in post-injury RGC survival and axon regeneration, mediated by subtype-specific neurotransmitter signaling.

### AC-derived zinc

In addition to neurotransmitters, vesicular zinc ion (Zn^2+^) serves as another rapidly reorganized transsynaptic signal from ACs to injured RGCs. ACs were initially found to inhibit RGC axon growth when co-cultured with them [100]. To investigate the inhibitory factors from ACs, Li et al. observed a rapid increase in zinc transporter 3 (ZnT3) in ACs, accompanied by elevated Zn^2+^ levels as early as one hour post-injury in the IPL, followed by accumulation in the GCL by day 3 (Table 1). This pathological Zn^2+^ translocation impairs RGC survival and regenerative capacity, effects that are reversed by Zn^2+^ chelation [15, 101] or AC-specific ZnT3 knockout [102]. These findings reveal vesicular Zn^2+^ as key transsynaptic inhibitor of neuroprotection and axon regeneration (Fig. 2D).

Regarding the source of Zn^2+^, preliminary findings suggest that nitric oxide (NO), generated by a subset of ACs, contributes to the elevation of Zn^2+^ levels following ONI. In addition, BCs may also play a role in pathological Zn^2+^ accumulation. After injury, glutamate released from BCs could activate ACs via NMDA receptors on their postsynaptic membranes, triggering depolarization, increased activity, and subsequent Zn^2+^ accumulation [94, 103].

Taken together, these findings indicate that synaptic signals from retinal interneurons are dynamically reshaped following RGC axon injury and profoundly influence RGC survival and axon regeneration (Fig. 2).

**Table 1:** Summary of synaptic signal dysregulation following RGC axon injury and pertinent insults

Synaptic signal	Alteration	Species	Model/ Disease	Time post-injury initiation	Specimen	Reference
Glutamate	Increased	Rat	ONI	3–7 days	Aqueous humor	[59]
		Rat	ONI	1–28 days	Vitreous body	[60]
		Dog	Glaucoma	Not specified	Vitreous body	[58]
		Monkey	OHT	Varying (126–226 days)	Vitreous body	[57]
		Human	Glaucoma	Not specified	Vitreous body	[57]
	Unchanged	Rat	OHT or ONI	1 day − 9 weeks	Vitreous body	[61]
		Monkey	CRAO	6 hours	Vitreous body	[63]
		Monkey	OHT	Varying (20 days − 13 months)	Vitreous body	[62, 64]
		Human	Glaucoma	Not specified	Vitreous body	[65]
GABA and glycine	Increased (AC activity)	Mouse	ONI	1–7 days	Retina	[16]
Dopamine	Decreased (DAC activity)	Mouse	ONI	1–7 days	Retina	[17]
Zn^2+^	Increased	Mouse	ONI	1 hour – 3 days	Retina	[15]
		Human	Glaucoma	Not specified	Aqueous humor	[104, 105]

Abbreviations: AC, amacrine cell; CRAO, central retinal artery occlusion; DAC, dopaminergic amacrine cell; GABA, γ-aminobutyric acid; OHT, ocular hypertension; ONI, optic nerve injury; Zn^2+^, zinc ion

### Synaptic modulation of intrinsic mechanisms in RGCs

Synaptic signals regulate neuronal activity and activate signal transduction cascades within RGCs, thereby modulating intrinsic mechanisms that govern cell survival and axon regeneration. In this section, we summarize recent advances in understanding how these signals regulate intrinsic repair programs in RGCs following injury.

#### Signaling pathways

Synaptic signals recruit intracellular signaling pathways that are critical determinants of RGC fate following axon injury. A prominent example is mammalian target of rapamycin (mTOR) pathway, which is well-established as a pro-regenerative mechanism [106–108]. Light stimulation, essential for melanopsin overexpression-mediated axon regeneration, enhances mTOR signaling, whereas light deprivation abolishes both mTOR pathway activation and axon regrowth [89]. Notably, mTOR pathway activation is also detected in nearly all regenerating RGCs after a combinatorial treatment of AC silencing and IGF1 overexpression [16]. Zn^2+^-targeted interventions also converge on this pathway, as Zn^2+^ elimination via chelation or ZnT3 knockout enhances phosphorylated S6 levels in RGCs [109]. While mTOR pathway serves as downstream regulator of synaptic signals, it also promotes dendritic maintenance or regeneration, which in turn may reinforce excitatory synaptic connectivity [11, 12, 14].

Other signaling pathways are also involved in mediating the effects of synaptic signals following axotomy. For instance, enhancing dopaminergic signal boosts both CaMKII/CREB and cAMP signaling, while downregulating the dual leucine zipper kinase (DLK)/c-Jun pathway in RGCs [17]. Interestingly, activation of CaMKII/CREB pathway [110, 111] and inhibition of DLK/c-Jun cascade [112] are both associated with improved RGC survival but reduced axon regeneration. In contrast, cAMP signaling is essential for both RGC survival and neurite extension [113, 114]. Additionally, Zn^2+^ chelation not only attenuates apoptotic signaling but also suppresses CHOP pathway [15], which is also rapidly upregulated after ONI and contributes to RGC degeneration [115].

Importantly, several studies have demonstrated that simultaneously targeting both synaptic signals and intrinsic signaling pathways yields enhanced pro-regenerative effects [15, 17, 101], However, the molecular crosstalk between these extrinsic and intrinsic mechanisms remains poorly understood and warrants further investigation.

#### Transcriptional and epigenetic regulation

Transcriptional and epigenetic regulation represent key intrinsic targets for axon regeneration [116]. Altered synaptic signals activate signaling pathways such as CaMKII/CREB, which initiate the transcription of multiple downstream effectors [110, 111]. Neuronal activity itself is capable of inducing gene expression, a phenomenon known as excitation-transcription coupling [117]. Notably, AC-targeted ZnT3 knockout leads to transcriptional shifts in RGCs, altering expression of genes involved in synaptic connection, neural cell fate and growth [102].

Even less is known about the epigenetic mechanisms underlying transsynaptic mechanisms in regeneration. One study demonstrated that electrical stimulation facilitates neurite outgrowth via ten-eleven translocation (TET), a DNA demethylase [96]. Additionally, our preliminary findings suggest histone acetylation as a key mechanism downstream of Zn^2+^-related treatment [118]. However, the transcriptional and epigenetic programs that mediate synapse-driven neuroprotection and axon regeneration remain to be fully elucidated.

#### Metabolic and oxidative stress

Synaptic signals can also affect cellular metabolism and oxidative stress in RGCs. For instance, elevated Zn^2+^ levels induce structural damage to RGC mitochondria, triggering mitochondrial-mediated integrated stress response, thereby contributing to ONI-induced RGC death [119]. Conversely, Zn^2+^ elimination reduces reactive oxygen species production after ONI by activating the Nrf2 pathway, thereby preserving RGCs and promoting axon regeneration [120].

Collectively, these findings indicate that altered synaptic signals reshape the intrinsic programs of lesioned RGCs, accounting in part for their prominent roles in neuroprotection and axon regeneration. However, a comprehensive integration of synaptic signal-driven pathways and intrinsic repair mechanisms after RGC axon injury is still lacking. In-depth analysis of this molecular crosstalk is essential for the rational design of combinatorial therapies.

## Mechanisms that reshape interneuron-to-RGC signals

Although signals from ACs and BCs are rapidly altered following RGC axon injury and become critical regulators of neuroprotection and axon regeneration, these interneurons do not extend processes into the optic nerve, where injury to RGC axons occurs. This raises a fundamental question: how do they respond to a lesion that is anatomically remote? To date, few studies have directly addressed this issue. In this section, we synthesize current evidence on how an axotomized neuron relays information about axonal injury to its presynaptic partners within the nervous system, with the goal of stimulating discussion and encouraging further investigation into the mechanisms by which retinal synaptic signaling becomes dysregulated following RGC axon lesion. Briefly, signals generated at the injury site propagate along the axon to the soma, and subsequently influence presynaptic interneurons via transsynaptic communication, ultimately triggering pathophysiological changes in their activity and signaling.

### Axon-to-soma signals that remodel transsynaptic communication

Traditionally, axon-to-soma signaling has been studied from an intrinsic perspective, emphasizing how axon insults activate transcriptional and epigenetic responses to support regeneration [121]. Recent studies, however, have revealed new roles of retrograde injury signaling in transsynaptic communication.

**Fig. 3:**
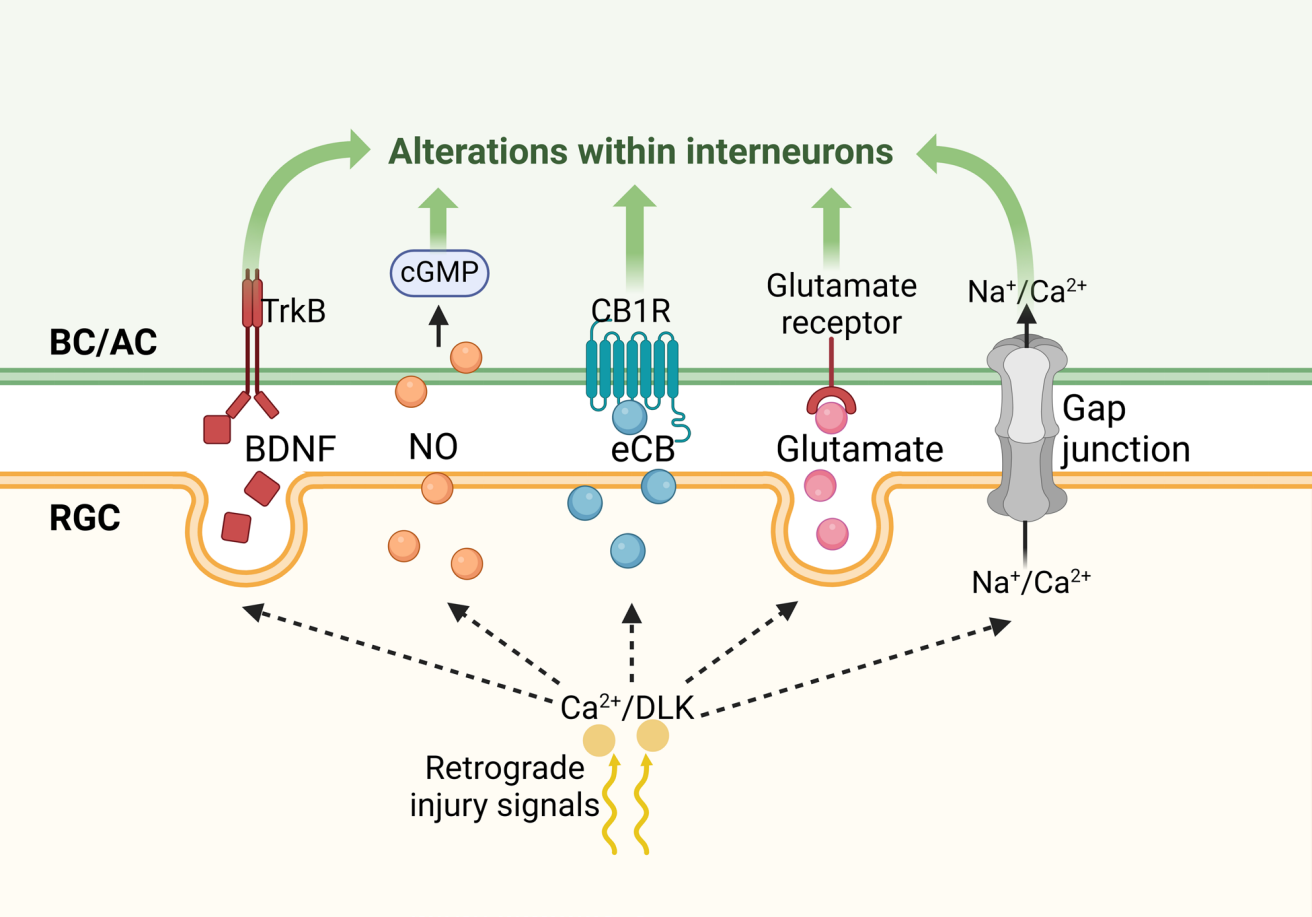
Potential mechanisms by which synaptic signals are reshaped after RGC axon injury. Retrograde injury signals, such as Ca^2+^ and DLK, propagate from axon to soma after axon injury. Most retrograde messengers are released in a Ca^2+^-dependent way. Local postsynaptic Ca^2+^ elevation potentially trigger retrograde transsynaptic signaling, including the release of postsynaptic BDNF, NO, eCB, glutamate and gap junctions-mediated signaling. These are important candidates for transsynaptic retrograde signals that mediate synaptic dysregulation after axon injury. Abbreviations: AC, amacrine cell; BC, bipolar cell; BDNF, brain-derived neurotrophic factor; Ca^2+^, calcium ion; CB1R, cannabinoid receptor 1; DLK, Dual leucine zipper kinase; eCB, endocannbinoid; Na^+^, sodium ion; NO, nitric oxide; RGC, retinal ganglion cell; TrkB, tropomyosin-related receptor kinase-B. Created in https://BioRender.com

Calcium ion (Ca^2+^), a well-established mediator of axon-to-soma signaling, has recently emerged as a critical player in transsynaptic communication. The rapid back-propagation of calcium wave from the axon to the soma following axon injury has been documented in various animal models, both in the peripheral nervous system (PNS) and the CNS [122–125]. Notably, this back-propagating calcium signal travels at approximately 10 μm/s in cultured dorsal root ganglion neurons and is estimated to reach around 1.5 μm/s in vivo [124], making it a potential upstream signal for the rapid alterations in synaptic signals observed after RGC axon injury. Intriguingly, Nagendran and Taylor demonstrated that calcium release from intracellular stores after axotomy drives dendritic spine loss, hyperexcitability and synaptic disinhibition [126], implicating retrograde calcium signaling in transsynaptic plasticity (Fig. 3). While calcium signaling is known to influence both neuronal survival and axon degeneration [111, 127, 128], its specific role in modulating RGC-interneuron communication remains to be elucidated.

DLK is another key axon-to-soma injury signal that influences transsynaptic communication. It is rapidly activated following axon injury via a Ca^2+^/cAMP/PKA signaling cascade [123, 129], and is essential for initiating retrograde injury signaling [112, 130, 131]. As previously discussed, DLK is a well-established mediator of axotomy-induced apoptosis [132–134], yet it is required for axon regeneration [112]. After ONI, DLK coordinates retrograde signaling that drives RGC degeneration [112, 135], a process dependent on palmitoylation of DLK [136, 137]. Beyond its established role in injury-induced degeneration, DLK has conserved functions in synaptic development, neuronal stress responses, and neuroimmune interactions [138, 139] (and reviewed in [140, 141]), and has recently been identified as a critical regulator of upstream synaptic loss following motoneuron axotomy [54]. These findings highlight DLK’s role in orchestrating injury responses that extend beyond the injured neuron to reshape broader synaptic connectivity.

Once injury signals reach the soma, they must then traverse the synaptic cleft to reach presynaptic interneurons.

### Potential transsynaptic retrograde messengers that contribute to synaptic signal dysregulation

The signaling factor responsible for transmitting injury cues from a lesioned postsynaptic RGC to its presynaptic partners remains largely uncharacterized. Notably, retrograde messengers dynamically regulate CNS development, synaptic maturation and plasticity throughout life [142]. We propose that post-axotomy transsynaptic retrograde signaling may parallel, at least in part, mechanisms observed in developmental and physiological contexts. Besides the fundamental properties of CNS developmental or physiological retrograde messengers [20], a pathophysiological retrograde injury messenger would be characterized by two additional features:

1. Under physiological conditions, the synthesis and release of retrograde messenger are maintained at a stable level; following axon injury, its production or release either increase (positive messenger) or decrease (negative messenger).
2. The messenger, either independently or in conjunction with other factor(s), affects RGC survival or axon regeneration.

As discussed, synaptic signals critically influenced the fate of injured RGCs, shaping both survival and regenerative capacity. If a retrograde messenger alters interneuron-to-RGC signaling following after injury, it likely plays a pivotal role in determining neuronal survival and axon regrowth. Here, we summarize candidate retrograde messengers that may mediate post-lesional signaling from RGCs to presynaptic interneurons. For clarity and brevity, we highlight representative examples rather than an exhaustive list. Notably, most of these retrograde messengers are released in a calcium-dependent manner [20], suggesting a coordinated signal cascade initiated by axon-to-soma injury communication (Fig. 3).

#### Brain-derived neurotrophic factor

Brain-derived neurotrophic factor (BDNF) is a prototypical neurotrophin known to retrogradely modulate presynaptic function [143, 144]. Impaired retrograde axonal transport of BDNF and its cognate receptor, tropomyosin-related receptor kinase-B (TrkB), from the brain to RGC somas has been implicated in the progressive and selective degeneration of the ganglion cell layer and optic nerve in glaucomatous models [145, 146]. Following ONI, intraocular BDNF application exerts a neuroprotective effect [147–149], and BDNF deficiency in heterozygous mutants exacerbates inner retinal degeneration in a glaucomatous model [150]. Conversely, overexpression of BDNF or TrkB enhances RGC survival following ONI [151]. These studies underscore the pivotal role of BDNF/TrkB signaling in promoting RGC neuroprotection after axon injury.

However, much existing evidence lacks cell-type specificity. Recent immunofluorescent and transcriptomic studies have revealed that TrkB is expressed not only in RGCs but also in retinal interneurons that synapse onto them [152–154]. This broader expression pattern raises the possibility that BDNF may mediate transsynaptic communication within the retinal circuit after injury. Further investigation is needed to determine whether BDNF acts as a retrograde messenger that modulates synaptic signals to lesioned RGCs.

#### Nitric oxide

Nitric oxide (NO) is a hydrophobic gasotransmitter that readily diffuses through biological membranes and plays a pivotal role in retrograde signaling during CNS development and physiological processes [155, 156]. For instance, NO contributes to retrograde spread of long-term depression in the developing visual pathway [156]. It is mainly generated by the nitric oxide synthase (NOS) family, whose expression and activity are tightly regulated throughout development. In Drosophila brain, downregulation of NOS activity at the onset of axon remodeling correlates with NO’s role in promoting axon pruning and inhibiting axon regrowth [157].

Following ONI, NO may similarly participate in retrograde injury signaling and the remodeling of synaptic signals. Supporting this notion, goldfish RGCs exhibit increased neuronal NOS expression and activity after axotomy, with NO-cGMP signaling promoting neurodegeneration [158]. In adult hamsters, NOS inhibition confers dose-dependent neuroprotection following unilateral ONI [149].

While the precise molecular mechanisms remain to be fully elucidated, NO emerges as a significant candidate for mediating transsynaptic injury signaling.

#### Endocannabinoids

Lipid-derived molecules serve as retrograde messengers in the CNS, with the endocannabinoid (eCB) system representing one of the most well-established transsynaptic retrograde signaling mechanisms [159]. By differentially modulating glutamatergic and GABAergic transmission, eCBs regulate presynaptic neurotransmitter release [160].

eCBs also confer neuroprotection against excitotoxicity. For instance, ischemia-reperfusion injury results in enhanced activity and expression of fatty acid amide hydrolase, an eCB-degrading enzyme, accompanied by a significant reduction in endogenous eCB levels [161]. Activation of CB1 receptor has been shown to mitigate excitotoxic damage [162, 163].

Interestingly, anandamide, a key eCB, inhibits motor neuron axon regeneration in C. elegans [164], whereas in mammalian PNS, eCBs promote postlesional regeneration [165]. Given their established role in retrograde transsynaptic signaling, eCBs represent a compelling class of molecules for further investigation as mediators of transsynaptic injury communication.

#### Glutamate

Glutamate can also function as a retrograde signaling molecule in the CNS [166]. As noted above, its levels might increase after RGC axon injury. However, identifying the cellular source of this elevation remains challenging due to intricate regulation of extracellular glutamate. Employing cell type-specific gene tools to manipulate glutamate release, reuptake, or receptor activation would provide novel insights into glutamate’s potential role in post-injury synaptic signal remodeling.

#### Gap junction

Electrical synapses, mediated by gap junctions (GJs), enable dynamic and bidirectional communication between coupled neurons [167]. Various forms of electrical coupling exist among retinal neurons [168], including between RGCs and GABAergic ACs [169]. Tracer injections into genetically labeled intrinsically photosensitive RGCs (ipRGCs) have revealed their coupling with diverse AC subtypes [170, 171], some of which express serotonin, NOS or neuropeptide Y [171]. Notably, recent studies suggest a biased directionality in ipRGC-to-AC transmission, indicating ipRGCs exert feedback regulation on AC activity [172]. Furthermore, pharmacological inhibition or genetically ablation of GJs confers a neuroprotective effect in a glaucomatous model [173]. Together, these findings underpin the significance of GJ-mediated retrograde communication in physiological and pathophysiological conditions.

Collectively, insights into axon-to-soma and transsynaptic retrograde messengers may guide the discovery of RGC-to-interneuron injury signaling. Such understanding could help explain how presynaptic retinal interneurons respond to RGC axon damage, and potentially reveal novel therapeutic targets.

## Glial modulation of interneuron-to-RGC signals

Glial cells, including microglia, astrocytes, and Müller cells, play a key role in regulating synapses between interneurons and RGCs [21]. Following axon injury, they dynamically modulate synaptic signals.

### Microglial cells

Microglia are key regulators of synaptic refinement in the CNS and actively remodel interneuron-to-RGC synapses. These cells contribute to synaptic remodeling in both the developing retina and brain, likely through complement-dependent mechanisms, although other pathways may also contribute (reviewed in [174]). The developmental upregulation of complement components C1q and C3 marks synaptic structures for elimination [175]. Microglia then selectively recognize C3 via complement receptor 3 (CR3), which triggers phagocytosis of the targeted synaptic elements [176]. In various CNS disorders, synapse elimination by microglia becomes dysregulated (reviewed in [174, 177]).

**Fig. 4:**
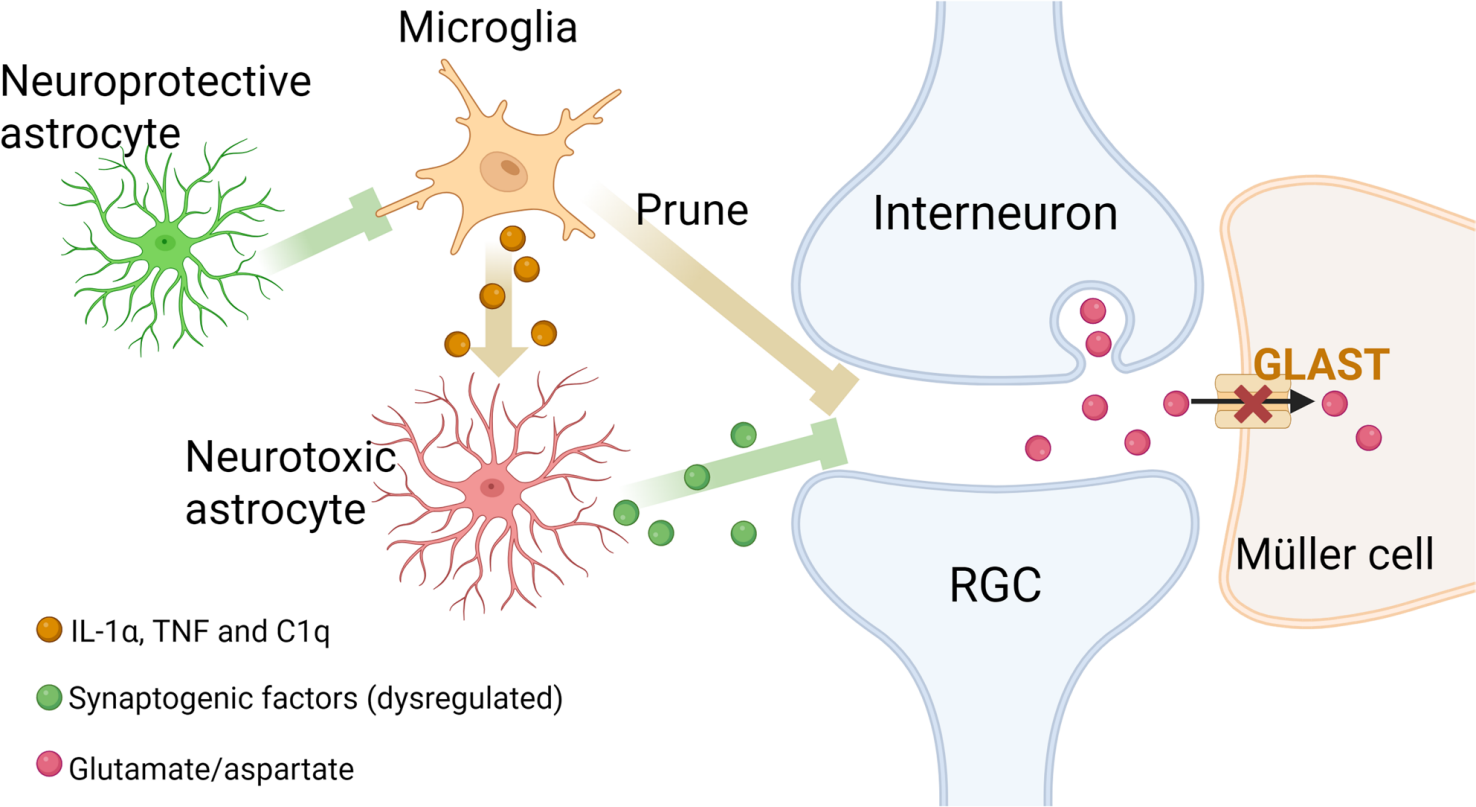
Glial cells modulate interneuron-to-RGC synapses following injury. Microglial cells prune retinal interneuron-to-RGC synapses following RGC axon damage. They also secrete IL-1α, TNF and C1q to induce neurotoxic astrocytes, which inhibit synapse formation via dysregulated synaptogenic factors. Neuroprotective astrocytes prevent microglial activation, thereby inhibiting neurotoxic astrocyte activation. After retinal ischemia or elevated intraocular pressure, GLAST-mediated Müller uptake of excitatory amino acids is impaired, leading to extracellular accumulation of glutamate or aspartate. Abbreviations: C1q, complement component 1, subcomponent q; GLAST, glutamate/aspartate transporter; IL-1α, interleukin 1 alpha; RGC, retinal ganglion cell; TNF, tumor necrosis factor. Created in https://BioRender.com

Similarly, after RGC axon injury, synapses between interneurons and RGCs undergo dynamic microglia-mediated remodeling. In a genetic model of OHT, microglia have been shown to engulf RGC postsynaptic components within the IPL in a C1q-dependent manner [178]. Consistently, acute OHT also triggers microglial pruning of C1q-tagged RGC dendrites [179]. Following ONI, microglia prune synapses in the IPL, and activation of microglial Sirtuin 1 hampers this process and protects RGCs from degeneration [180] (Fig. 4). However, it is also suggested that microglia may not directly contribute to RGC degeneration or axon regeneration after ONI [181]. Further research is necessary to clarify their role in transsynaptic communication following RGC axon injury and how this impacts neuroprotection and axon regeneration.

### Astrocytes

Astrocytes regulate synaptic elimination, formation, and function throughout the CNS [182]. They prune synapses during development and in the mature brain [183], and secrete essential synaptogenic factors, such as thrombospondins [184], secreted protein acidic and rich in cysteine-like 1 (SPRACL1) [185], glypicans 4 and 6 [186]. Notably, glypicans 4 and 6 are able to induce synapse formation between cultured RGCs [186]. In addition, astrocytes support synaptic energy metabolism by bridging blood vessels and neurons [187, 188], and astrocyte-derived glutamate can enhance excitatory synaptic strength [189]. These findings underscore their multifaceted role in synaptic modulation.

Astrocytes also regulate synaptic signals following axon injury. After ONI, they are converted to a reactive state by microglia-derived cytokines interleukin 1α (Il-1α), tumor necrosis factor (TNF) and C1q, and acquire a neurotoxic phenotype that contributes to RGC loss [190]. These reactive astrocytes exhibit altered expression of synaptogenic factors, including decreased glypican 6 and SPARCL1, and increased glypican 4, thrombospondins, resulting in the formation of fewer and weaker synapses between cultured RGCs compared to resting astrocytes [190]. Furthermore, the induction of neurotoxic astrocytes is required for RGC degeneration in both ONI and OHT models, and preventing astrocyte activation not only preserves RGCs but also protects their dendrites and synaptic function [191]. Cameron et al. further showed that neuroprotective astrocytes can suppress microglial activation, thereby preventing generation of neurotoxic astrocytes and promoting RGC survival [192] (Fig. 4). The extent to which astrocyte subtypes and broader glial networks regulate interneuron-to-RGC synapses following RGC axon injury remains an important area for future investigation.

### Müller cells

Müller cells span the entire thickness of the neuroretina and envelop synapses within the IPL. They regulate synaptic transmission by taking up and metabolizing neurotransmitters such as GABA and glutamate [193], releasing gliotransmitters [21] and protecting RGCs from glutamate-induced excitotoxicity [194]. Additionally, Müller cells produce BDNF in vitro [195, 196], suggesting they may contribute to synaptic plasticity and neuroprotection.

Under pathological conditions such as ischemia and elevated intraocular pressure, GLAST-mediated Müller uptake of excitatory amino acids can be compromised [197, 198] (Fig. 4). This dysfunction can lead to accumulation of extracellular glutamate, promoting excitotoxicity and potentially altering interneuron-RGC synaptic signaling.

In summary, glial cells are key regulators of transsynaptic communication in response to axon injury. Their diverse roles in synapse remodeling, trophic support, and neurotransmitter homeostasis indicate a potentially significant yet incompletely understood impact on synaptic signal alterations following RGC axon injury. Further investigation is required to delineate the complex interactions between glial cells and synapses in pathophysiological conditions such as ONI, and to explore their therapeutic potential.

## Clinical translation towards biomarkers and synapse-targeted therapeutics

The translation of aforementioned transsynaptic mechanisms into clinically viable biomarkers and therapeutic strategies represents an emerging frontier in visual restoration. While the critical role of these signals is well-established in animal models, their clinical significance remains a pressing question. Specifically, can dysregulated synaptic signals serve as biomarkers of human diseases involving axonal damage, and can they be therapeutically harnessed? This potential is powerfully exemplified by glaucoma, the leading cause of irreversible blindness worldwide that is characterized by progressive optic neuropathy [199]. In line with early post-injury degeneration of RGC dendrites, ongoing efforts aim to identify structural biomarkers within the IPL using retinal imaging to improve glaucoma diagnosis and longitudinal disease monitoring [200]. Complementing imaging approaches, analysis of intraocular specimens, such as aqueous humor and vitreous body, provides a valuable window into pathological processes of glaucoma (Table 1). For instance, glutamate levels may be elevated in the vitreous of both glaucomatous non-human primates and humans [57]. More recently, increased zinc levels have been identified in the aqueous humor of glaucoma patients [104, 105], robustly supporting zinc’s dual potential as both a biomarker for disease and a promising therapeutic target.

On the therapeutic front, directly targeting synaptic signaling has proven challenging. As mentioned in section “BC-derived glutamate”, two clinical trials reported no significant slowing of disease progression in patients with open-angle glaucoma after 48 months of oral memantine treatment (NCT00141882, NCT00168350) [87]. Still, another orally administered synaptogenic small molecule SPG302, which is currently under clinical evaluation for amyotrophic lateral sclerosis (ALS, NCT05882695), has shown to reverse synaptic pathology and protects RGCs in a chronic OHT model, suggesting potential for future neuroprotective translation in glaucoma [201]. Although the anatomical isolation of the retina (Fig. 1A) presents challenges for drug access, it also enables highly targeted interventions. Recent advances in localized ocular delivery further support this outlook by accelerating the development of synapse-targeted therapies. Nanoparticle-based approaches, for instance, now enable the sustained intraocular release of therapeutics, such as zinc chelators, showing promise in pre-clinical models [202, 203]. Concurrently, the field of ophthalmology is at the forefront of therapeutic innovation, with clinical studies actively pioneering in vivo gene therapy in human patients [204]. Furthermore, stem cell-based approaches, which have demonstrated potential for addressing interneuron deficits in CNS disorders like Parkinson’s disease and epilepsy [205], could be adapted to correct dysregulated synaptic transmission in the retina. The convergence of these advanced technologies significantly enhances the translational potential of synapse-related targets identified in rodent models. Ultimately, applying these strategies offers a multifaceted path towards protecting RGCs, preserving axonal integrity, and preventing irreversible vision loss.

## Future directions

Synaptic regulation of RGC survival and axon regeneration has been supported by structural evidence [10–14] and further characterized at molecular level [15–18]. However, it remains unclear whether dendritic degeneration after axon injury is directly detrimental to RGC survival and, if so, which molecular mediators underlie this effect (section “RGC dendrites”). To more precisely delineate how synaptic dysfunction contributes to RGC degeneration, the field would benefit from an integrated framework that links synaptic architecture to transsynaptic signaling mechanisms. For example, building on dendrite-regenerating interventions (section “RGC dendrites”; Fig. 1D), one could pharmacologically or genetically manipulate the production and release of a defined synaptic signal in interneurons, neutralize the signaling molecule within the synaptic cleft, or modulate its receptors on the postsynaptic RGC membrane. Such strategies would allow rigorous testing of whether specific forms of neurotransmission or neuromodulation promote RGC survival following dendritic regeneration.

Direct in vivo monitoring of transsynaptic communication and downstream RGC responses would further enable investigators to dissect post-axotomy retinal synaptic dysregulation and quantify how synaptic signaling and resulting electrophysiological/electrochemical events are reshaped by synapse-targeted interventions. Yet, such measurements remain technically challenging. Encouragingly, recent advances in in vivo imaging of neurotransmitters and neuromodulators (reviewed in [206]), together with microelectrode array technologies (reviewed in [207, 208]), provide a powerful toolkit to address these questions. In addition, clinical evidence, including synapse-related structural and molecular biomarkers, as well as outcomes from synapse-targeted therapies in patients with glaucoma, holds substantial translational promise and may also yield critical mechanistic insights.

Another unresolved question in transsynaptic pathobiology is how RGCs relay axonal injury signals back to their dendrites and onward to presynaptic retinal interneurons. The rapid synaptic remodeling observed after axon damage (Table 1) implies early injury-signal transfer both within individual neurons (from axon to soma) and across synaptically connected cells (from RGCs to interneurons). In other regions of the nervous system, post-axotomy Ca^2+^ signaling and DLK pathways have been implicated as key coordinators of axon-to-soma injury signaling and synaptic remodeling (section 3.1). At the glial-synaptic interface, transmembrane molecules such as MHC-I (Box 1), as well as secreted factors, including complement components, tumor necrosis factors and interleukins (section 4.1–4.2), are also of particular interest. Nevertheless, given the diversity of retinal neuron types and the complexity of retinal synaptic organization, the molecular pathways that orchestrate transsynaptic responses to RGC axon injury require careful and systematic investigation.

Axon injury is a hallmark of various CNS disorders, underscoring the broad relevance of identifying synapse-driven protective and pro-regenerative cues across neurodegenerative and neurotraumatic disease contexts. Historically, the inherent complexity of CNS circuitry has hindered efforts to decipher transsynaptic interactions that govern neuroprotection and regeneration. Recent advances in transcriptomic technologies [209–211] and synaptomic approaches [212–214], however, have substantially improved our ability to map and integrate the molecular, structural and functional organization of neuronal networks. Systemic application of these tools to characterize disease-associated synaptic alterations across diverse CNS conditions may provide a roadmap for elucidating transsynaptic mechanisms of neural injury and for identifying therapeutic targets that promote neural repair.

## Conclusion

RGCs reside within a complex cellular ecosystem comprising multiple glial and immune cells, neighboring neurons, and extracellular matrix. Complementing the established literature that emphasizes RGC intrinsic growth pathways and RGC-microenvironment interactions in neuroprotection and axon regeneration, an emerging body of work sheds light on the importance of synaptic communication. In this context, synaptic connections between retinal interneurons and RGCs undergo pronounced structural and molecular remodeling in response to RGC axon injury. These synaptic alterations regulate RGC survival and axon regeneration, at least in part by engaging intrinsic stress-response and repair programs. Meanwhile, several limitations point to key future directions. These include the need for integrated structural-molecular evidence, deepened mechanistic insights, clinical validation and assessment of how broadly synapse-mediated neuroprotection and regenerative signaling generalize across the CNS.

## Data Availability

No datasets were generated or analysed during the current study.

## Abbreviations

AC: Amacrine cell
AMPK: Adenosine monophosphate-activated protein kinase
ALS: Amyotrophic lateral sclerosis
BC: Bipolar cell
BDNF: Brain-derived neurotrophic factor
C1q: Complement component 1, subcomponent q
C3: Complement component 3
CB1R: Cannabinoid receptor 1
CNS: Central nervous system
CR3: Complement receptor 3
DA: Dopamine
DAC: Dopaminergic amacrine cell
DLK: Dual leucine zipper kinase
EAAC1: Excitatory amino acid carrier 1
eCB: Endocannabinoid
GABA: γ-Aminobutyric acid
GCL: Ganglion cell layer
GJs: Gap junctions
GLAST: Glutamate/aspartate transporter
IGF1: Insulin-like growth factor 1
INL: Inner nuclear layer
ipRGCs: Intrinsically photosensitive retinal ganglion cells
IPL: Inner plexiform layer
mTOR: Mammalian target of rapamycin
NMDA: N-methyl-D-aspartic acid
NO: Nitric oxide
NOS: Nitric oxide synthase
OHT: Ocular hypertension
ONI: Optic nerve injury
ONL: Outer nuclear layer
OPL: Outer plexiform layer
PNS: Peripheral nervous system
PS: Photoreceptor segment
REDD2: Regulated in development and DNA damage response 2
RGC: Retinal ganglion cell
RNFL: Retinal nerve fiber layer
RPE: Retinal pigment epithelium
SCI: Spinal cord injury
SPARCL1: Secreted protein acidic and rich in cysteine-like 1
TET: Ten-eleven translocation
TrkB: Tropomyosin-related receptor kinase-B
Ca²⁺: Calcium ion
Zn²⁺: Zinc ion
